# Effect of glycine betaine on chilling injury in relation to energy metabolism in papaya fruit during cold storage

**DOI:** 10.1002/fsn3.957

**Published:** 2019-02-17

**Authors:** Yonggui Pan, Shanying Zhang, Mengqi Yuan, Hanliang Song, Tian Wang, Weimin Zhang, Zhengke Zhang

**Affiliations:** ^1^ College of Food Science and Technology Hainan University Haikou China

**Keywords:** chilling injury, energy metabolism, energy status, glycine betaine, papaya fruit

## Abstract

“Zhongbai” papaya fruit were treated with 15 mmol/L glycine betaine (GB) and then refrigerated at 6°C for 40 days to study the influence of GB on chilling injury (CI) and possible mechanism associated with energy metabolism. The results exhibited that GB treatment remarkably reduced the CI severity as indicated by lower CI index during storage. GB treatment lowered electrolyte leakage and malondialdehyde content, which accounted for maintenance of membrane integrity and reduced lipid peroxidation. Moreover, GB treatment improved the energy status as revealed by increased adenosine triphosphate (ATP) level, energy charge, and activities of energy metabolism‐related enzymes including mitochondrial membrane H^+^‐adenosine triphosphatase (H^+^‐ATPase) and Ca^2+^‐adenosine triphosphatase (Ca^2+^‐ATPase), succinate dehydrogenase (SDH), and cytochrome C oxidase (CCO). The results indicate that enhanced chilling tolerance in papaya fruit by GB treatment during cold storage might be ascribed to improved energy status in association with increased activities of energy metabolism‐related enzymes.

## 1 INTRODUCTION

Papaya (*Carica papaya* L.) fruit is an important typical fruit with high economic value due to its rich nutritional constituents (carbohydrates, ascorbic acid, carotenoids, and papain) (Jing et al., 2015). Nevertheless, as a typical climacteric fruit, papayas following harvest undergo a rapid ripening and softening process within 6–8 days when stored at ambient temperatures (Pan, Yuan, Zhang, & Zhang, 2017). Refrigeration is recognized as one of the most effective technologies to slow the ripening and prolong the shelf life of harvested commodities (Aghdam & Bodbodak, 2013). Unfortunately, papaya fruit is liable to chilling injury (CI) when exposed to nonfreezing low temperatures below 12°C (Singh & Rao, 2005). Chilled papayas gradually appear CI symptoms as manifested by pitting, scald and shriveling of the peel, water‐soaking of the pulp, failure to ripening, loss of aroma and flavor, and high susceptibility to postharvest decay, which leads to severe decline in fruit quality and reduced marketability (Emond & Brecht, 2005). Although a few approaches, including modified atmosphere packing and hot water treatment, have been tested to attenuate CI of papaya fruit (Shadmani, Ahmad, Saari, Ding, & Tajidin, 2015; Singh & Rao, 2005), there is still an urgent requirement to explore more valid techniques for inhibiting or ameliorating CI of cold‐stored papayas.

Glycine betaine (GB), a quaternary ammonium compound, functions as a vital osmotic adjustment substance in plant cells, with higher level maintaining cellular solubility, conferring resistance to diverse abiotic stresses (Giri, 2011). It was reported that application of exogenous GB enhanced tolerance of *Arabidopsis thaliana* and *Solanum lycopersicum* plants against chilling stress (Karabudak, Bor, Özdemir, & Türkan, 2014; Xing & Rajashekar, 2001). Recent studies have also demonstrated that exogenous GB treatment may effectively dampen CI and improve postharvest quality in a variety of harvested crops, such as loquats (Sun, Jin, Shan, Jia, & Zheng, 2014; Zhang et al., 2016), bananas (Rodríguez‐Zapata et al., 2015), peaches (Shan et al., 2016; Wang et al., 2019), hawthorns (Razavi, Mahmoudi, Rabiei, & Aghdam, 2018), and zucchinis (Yao, Xu, Farooq, Jin, & Zheng, 2018). The mechanisms behind the observed CI alleviation by GB treatment mainly involve in enhancement of antioxidant capacity, activation of phenolic and sugar metabolisms, inhibition of membrane lipid degradation, as well as promotion of proline biosynthesis (Aghdam, Jannatizadeh, Luo, & Faliyath, 2018).

Energy is the foundation for sustaining life activities, and energy status profoundly affects diverse physiological processes in fruit (Jiang et al., 2007; Lin et al., 2018). Accumulating evidences suggest that cellular energy is indispensable for biosynthesis of membrane lipid, while adequate supply of adenosine triphosphate (ATP) may benefit in reducing loss of membrane integrity in fruit under chilling stress (Liu et al., 2011; Marangoni, Palma, & Stanley, 1996). Maintenance of energy level by various postharvest treatments has contributed to improved chilling tolerance in harvested crops, as demonstrated in peaches (Jin, Zhu, Wang, Shan, & Zheng, 2014; Jin et al., 2013), loquats (Jin et al., 2015), mangoes (Li, Zheng, Liu, & Zhu, 2014), bananas (Li, Limwachiranon, Li, Du, & Luo, 2016; Wang, Luo, Khan, Mao, & Ying, 2015), pears (Cheng, Wei, Zhou, Tan, & Ji, 2015), cucumbers (Chen & Yang, 2013), and bamboo shoots (Liu et al., 2016). The generation and utilization of energy depend on a series of energy metabolism‐related enzymes including H^+^‐ATPase, Ca^2+^‐ATPase, succinate dehydrogenase (SDH), and cytochrome C oxidase (CCO) (Jin et al., 2013). In a previous study, we noted that faster declines in ATP content and energy metabolism‐related enzymes activity occurred in papaya fruit during storage at 6°C when compared to those in cold‐stored fruit at 0°C, and accordingly, the more severe CI was observed in papaya fruit stored at 6°C (Pan et al., 2017). This finding indicates that there could be an intimate correlation between CI and energy metabolism in papaya fruit (Pan et al., 2017). However, to the best of our knowledge, there is a lack of information concerning the influence of GB on CI of papaya fruit and its possible mechanism involved in energy metabolism. The aim of this study was to evaluate whether GB could enhance chilling tolerance in papaya fruit via regulation of energy metabolism.

## 2 MATERIALS AND METHODS

### 2.1 Fruit materials and treatments

Physiologically matured papaya (*Carica papaya* L. cv. Zhongbai) fruit, with 9.67 ± 0.35% (*n* = 3) of soluble solids content and titratable acidity of 0.22 ± 0.006% (*n* = 3), were collected from a commercial orchard in Chengmai County, Hainan Province, China. Fruit were packed in plastic box and transported to the laboratory within 2 hr. The fruit with uniform size, color, and absence of mechanical damage and disease were chosen for the following experiment.

Papaya fruit were disinfected with 0.1% Sportak^®^ fungicide solution for 2 min, air‐dried and randomly assigned into two groups, with 170 fruit for each group. The first group was dipped in 15 mM GB (Aikeda Chemical Reagent Co., Ltd., Chengdu, China) solution for 10 min at room temperature, while the second group (control) was dipped in distilled water for 10 min. A GB concentration of 15 mM was screen as an optimum in terms of our preliminary experiment. After treatment with GB or water, the fruit were naturally air‐dried and stored at 6°C and 80%–90% relative humidity for 40 days. CI index and membrane permeability of papaya fruit were measured at 0, 10, 20, 30, and 40 days of storage. At the same interval during storage, the mesocarp tissues of fruit were taken and frozen in liquid nitrogen, and stored at −80°C until analysis. Each treatment contained three replications, the CI index was evaluated using 30 fruit for each replicate, and the other indicators were analyzed in 3 fruit per replicate.

### 2.2 CI index

Chilling injury index was measured using a rating scale on the basis of the proportion of chilling‐injured lesion area to the total surface area on each fruit (Zhang et al., 2017). Scale was categorized as follow: 0 = no signs of CI; 1 = less than 10% CI area; 2 = CI area ranging from 10% to 30%; 3 = CI area ranging from 31% to 50%; and 4 = more than 50% CI area. The CI index was calculated using the formula: CI index = ∑ (CI scale × number of fruit in each class)/(number of total fruit × 4) × 100%.

### 2.3 Electrolyte leakage and malondialdehyde (MDA) content

Cylindrical mesocarp tissues from three peeled fruit were taken using a cork borer and processed into 12 disks (5 mm diameter and 2 mm thickness for each) to measure electrolyte leakage using a conductivity meter according to the method described in Shadmani et al. (2015). MDA content was assayed with 3 g of mesocarp tissues using thiobarbituric acid (TBA) method as described in Wang, Li, Wang, and Li (2010). MDA content was expressed as nmol/g fresh weight (FW).

### 2.4 Determination of ATP, ADP, and AMP contents and energy charge

Adenosine triphosphate, ADP, and AMP were extracted according to the method of Yi et al. (2008) with some modifications. Two grams of mesocarp samples was homogenized with 6 ml of 0.6 M perchloric acid at 4°C for 20 min. The homogenate was centrifuged at 19,000 *g* for 20 min at 4°C. The supernatant was adjusted to pH 6.5–6.8 using 1 mol/L KOH, diluted to 5 ml with ultrapure water, and then passed through a 0.45‐mm hydrophilic membrane filter. The contents of ATP, ADP, and AMP were assayed by an Agilent 1260 high‐performance liquid chromatography (HPLC; Agilent Technologies Inc., Palo Alto, CA) equipped with a reversed‐phase Nova‐Pak C18 column and an ultraviolet (UV) detector. HPLC was executed using a procedure described in our previous report (Pan et al., 2017). The contents of ATP, ADP, and AMP were expressed as nmol/g FW. The energy charge was calculated as: Energy charge = ([ATP] + 1/2 [ADP])/([ATP] + [ADP] + [AMP]).

### 2.5 Measurement of energy metabolism‐related enzymes activity

Mitochondria were extracted from 20 g of papaya mesocarp tissues following our previous method described in Pan et al. (2017).

Activities of mitochondrial membrane H^+^‐ATPase and Ca^2+^‐ATPase were measured according to the release of phosphorus, as described in Li et al. (2016). One unit (U) of H^+^‐ATP and Ca^2+^‐ATPase activities was defined as the amount of enzymes needed for catalyzing the release of 1 μmol of phosphorus per minute.

Succinate dehydrogenase activity was measured using a SDH kit (Nanjing Jiancheng Bioeng. Inst., Nanjing, China) according to the instruction from the manufacturer. One unit (U) of SDH activity was defined as an increase of 0.01 in absorbance at 600 nm per minute. CCO activity was determined according to the method of Wang et al. (2015) with moderate modifications. The crude mitochondrial extract (0.2 ml) was added to the mixed solution that contained 0.2 ml reduced cytochrome C (2%, w/v) and 0.2 ml dimethyl phenylenediamine solution (20 mmol/L), and then was incubated at 30°C for 2 min. One unit (U) of CCO activity was defined as an increase of 0.01 at 510 nm absorbance per minute.

The activity of above‐mentioned enzymes was expressed as U/mg protein. Protein content of each enzyme was determined using the method of Bradford (1976).

### 2.6 Statistical analysis

All data were expressed as the means ± standard errors (*SE*). All data were subjected to the Independent Samples *t* test analysis for comparing the means using the SPSS 17.0 (SPSS, Inc., Chicago, IL). Asterisks (*) represent significant difference between the control and GB‐treated papaya fruit at the same day (*p <* *0.05).

## 3 RESULTS

### 3.1 CI index

**Figure 1:**
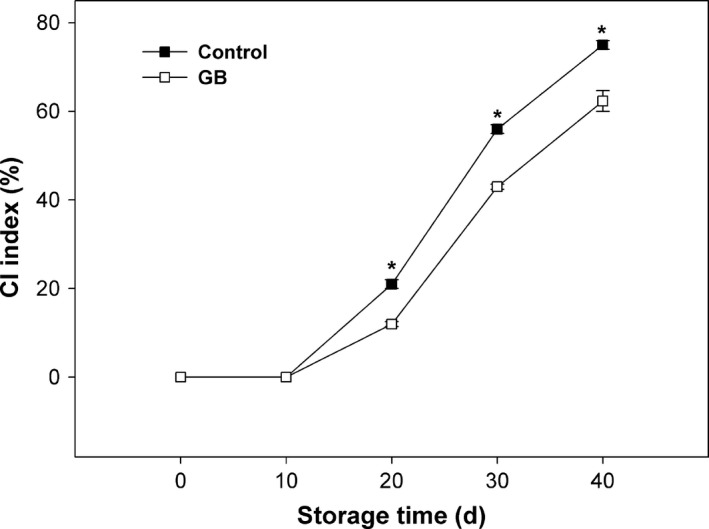
CI index in “Zhongbai” papaya fruit during cold storage at 6°C after treatment with 15 mmol/L GB and water (control). Data presented are means ± *SE* (*n* = 3). Asterisks represent a significant difference between the control and GB‐treated fruit at the each storage time (*p *<* *0.05)

As shown in Figure 1, CI symptoms in both control and GB‐treated papaya fruit did not appear within the initial 20 days of storage. Afterward, the CI severity in both control and GB‐treated fruit continuously increased over the rest of storage. Comparatively, CI index in GB‐treated fruit was significantly lower than that in control from 20 to 40 days of storage.

### 3.2 Electrolyte leakage and MDA content

**Figure 2:**
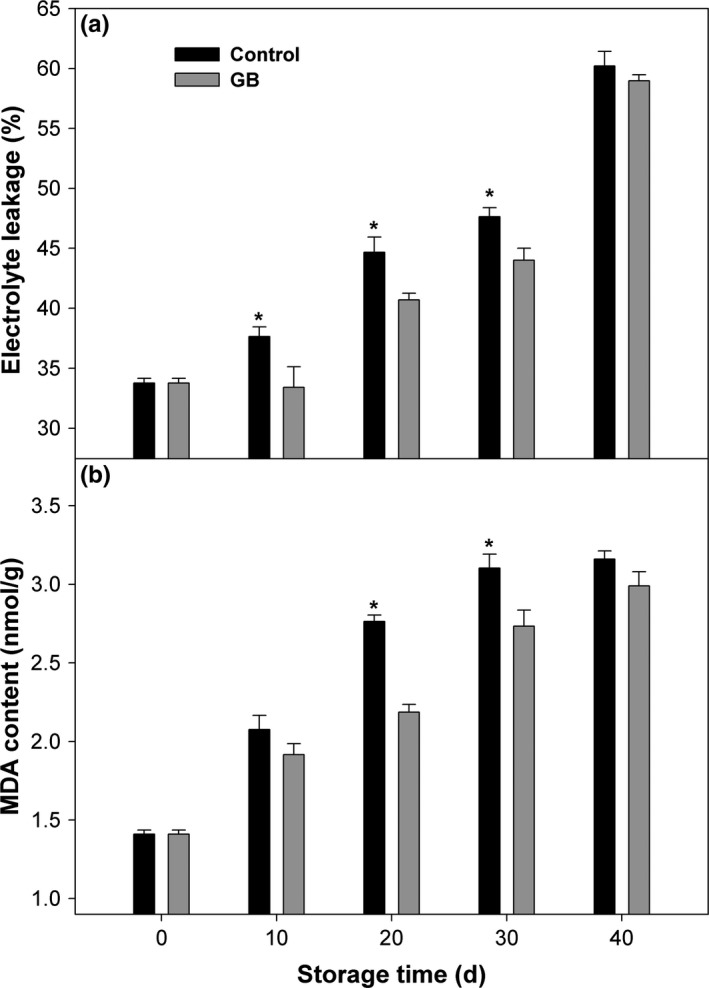
Electrolyte leakage (a) and MDA content (b) in “Zhongbai” papaya fruit during cold storage at 6°C after treatment with 15 mmol/L GB and water (control). Data presented are means ± *SE* (*n* = 3). Asterisks represent a significant difference between the control and GB‐treated fruit at the each storage time (*p *<* *0.05)

As depicted in Figure 2a, electrolyte leakage in control and GB‐treated fruit had a similar changing mode, with steadily increasing until end of storage. However, electrolyte leakage in GB‐treated fruit was lower than that in control fruit during the most of storage, with the exception of 40 days (Figure 2a). MDA content in control fruit sharply rose with the initial 30 days of storage and thereafter maintained at a stable level until end of storage (Figure 2b). GB treatment significantly inhibited the accumulation of MDA during the most of storage, except on 10 and 40 days of storage (Figure 2b).

### 3.3 Contents of ATP, ADP, and AMP and energy charge

**Figure 3:**
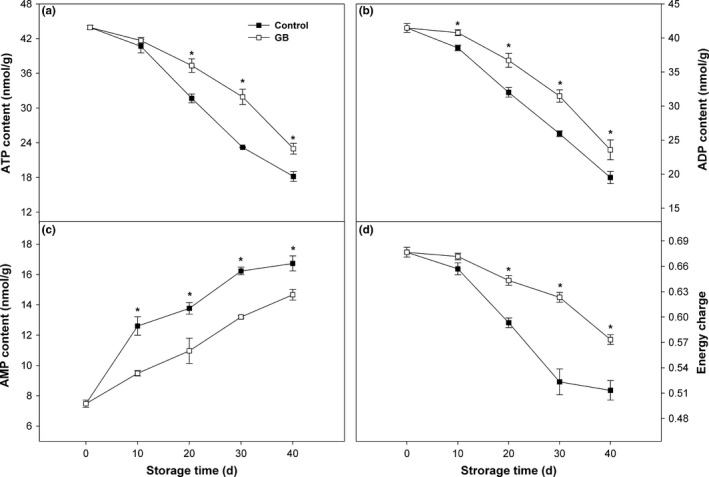
ATP (a), ADP (b), and AMP (c) contents and energy charge (d) in “Zhongbai” papaya fruit during cold storage at 6°C after treatment with 15 mmol/L GB and water (control). Data presented are means ± *SE* (*n* = 3). Asterisks represent a significant difference between the control and GB‐treated fruit at the each storage time (*p *<* *0.05)

Adenosine triphosphate and ADP contents in control fruit presented continuous downward trend as storage proceeded (Figure 3a,b). Decreasing trends of ATP and ADP contents were also occurred in GB‐treated fruit, but values of contents were significantly higher than those in control fruit during the most of storage period, except ATP content on 10 days (Figure 3a,b). AMP content in control fruit progressively increased throughout storage (Figure 3c). GB treatment inhibited the increase in AMP content, in which the AMP content in GB‐treated fruit was lower than that in control fruit throughout storage (Figure 3c). Energy charge in control fruit slightly decreased in the initial 10 days, sharply dropped from 10 to 30 days, and then held at a stable level over the remainder of storage (Figure 3d). Energy charge in GB‐treated fruit steadily decreased, but was significantly higher than that in control fruit during most of storage, except on 10 days (Figure 3d).

### 3.4 Mitochondrial membrane H^+^‐ATPase and Ca^2+^‐ATPase activities

**Figure 4:**
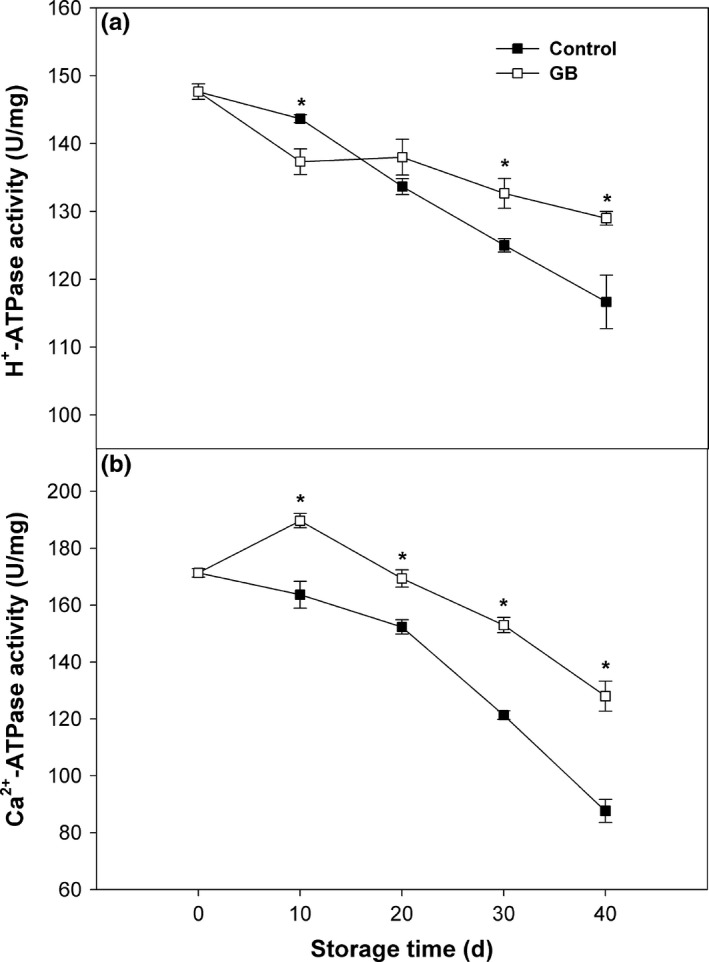
H^+^‐ATPase (a) and Ca^2+^‐ATPase (b) activities in “Zhongbai” papaya fruit during cold storage at 6°C after treatment with 15 mmol/L GB and water (control). Data presented are means ± *SE* (*n* = 3). Asterisks represent a significant difference between the control and GB‐treated fruit at the each storage time (*p *<* *0.05)

H^+^‐ATPase activity in control fruit showed a liner decreasing trend during storage (Figure 4a). Comparing with control fruit, H^+^‐ATPase activity in GB‐treated fruit was lower within the initial 10 days, but higher in the late stage of storage, typically on 30–40 days (Figure 4a). Ca^2+^‐ATPase activity in control fruit constantly declined throughout storage (Figure 4b). GB treatment stimulated an increase in Ca^2+^‐ATPase activity within the early storage (Figure 4b). After 10 days, Ca^2+^‐ATPase activity in GB‐treated fruit continuously decreased, but was higher than that in control fruit throughout storage (Figure 4b).

### 3.5 SDH and CCO activities

**Figure 5:**
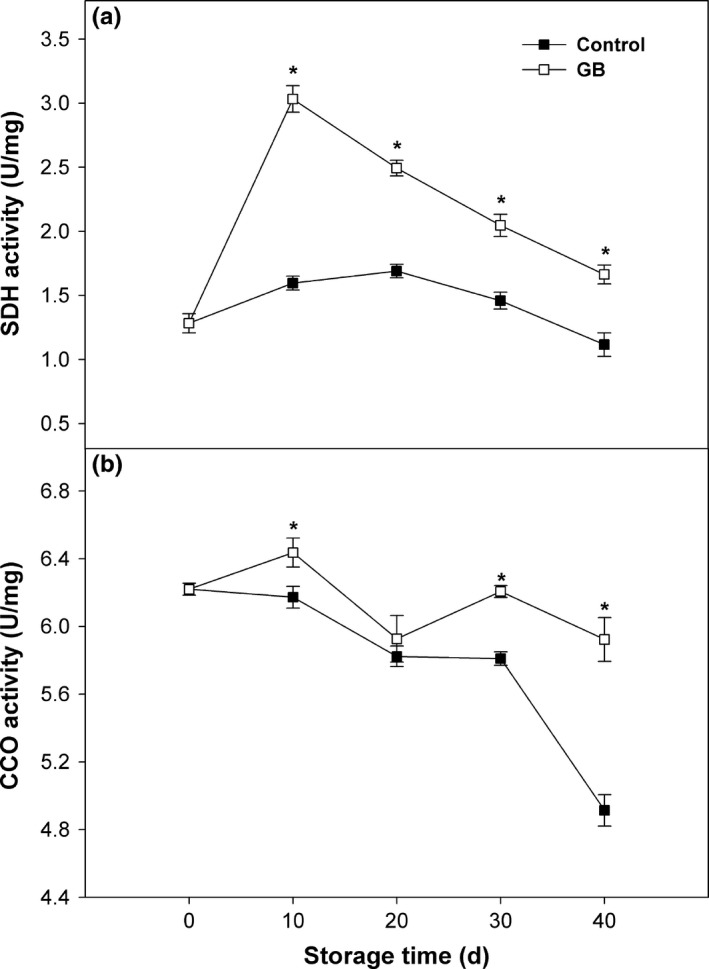
SDH (a) and CCO (b) activities in “Zhongbai” papaya fruit during cold storage at 6°C after treatment with 15 mmol/L GB and water (control). Data presented are means ± *SE* (*n* = 3). Asterisks represent a significant difference between the control and GB‐treated fruit at the each storage time (*p *<* *0.05)

Succinate dehydrogenase activity in control fruit slightly increased until midstorage (20 days) and then steadily decreased over the rest of storage (Figure 5a). SDH acidity in GB‐treated fruit presented a pattern of typical peak shape, with maximum appearing at 2 days of storage (Figure 5a). SDH activity in GB‐treated fruit maintained at higher level comparing with control fruit throughout storage (Figure 5a). CCO activity in control fruit decreased with a slow rate in the first 30 days, followed by a sharp decline until end of storage (Figure 5b). CCO activity in GB‐treated fruit showed a wave trend, but was higher than that in control fruit, with statistic difference exhibiting on 10, 30, and 40 days of storage (Figure 5b).

## 4 DISCUSSION

Chilling injury is a main factor restraining the quality of papaya fruit stored at low temperature (Pan et al., 2017). In the current study, CI symptoms including pitting, scald, water‐soaking, and decay were observed on the peel of refrigerated “Zhongbai” papaya fruit. GB is a pivotal osmotic regulating compound in higher plants, which can maintain membrane integrity, protect biomacromolecules function as well as confer resistance to various abiotic stresses (Mansour, 1998). In the present study, GB treatment by means of immersion at 15 mmol/L remarkably reduced the severity of CI in papaya fruit during 40 days of cold storage at 6°C. Similar inhibition of CI owing to GB treatment has also been observed in other harvested fruits such as loquats (Sun et al., 2014; Zhang et al., 2016), bananas (Rodríguez‐Zapata et al., 2015), peaches (Shan et al., 2016; Wang et al., 2019), and hawthorns (Razavi et al., 2018).

It is known that low temperature stress initiates membrane lipid phase transition from liquid crystalline to gelatin state as indicated by increased membrane permeability, which results in loss of membrane structure and function accompanying with CI development of fruit (Zhang et al., 2010). Process of membrane damage is generally involved in lipid peroxidation resulting from attack of overproduced reactive oxygen species (ROS), including ${\text{O}}_{2}^{-}$˙, H_2_O_2_, •OH, and ^1^O_2_ (Zhang et al., 2018). MDA is one of the low molecular weight end‐products of lipid peroxidation, and its level directly reflects the degree of oxidative damage to membrane (Dhindsa, Plumb‐Dhindsa, & Thorpe, 1981). Membrane permeability and MDA are usually employed to measure oxidative damage and membrane deterioration in response to chilling and other environmental stresses in plants (Zhang et al., 2017). In this study, electrolyte leakage and MDA overall increased along with CI development in papaya fruit during refrigeration, while GB treatment suppressed the increase in both parameters, indicating that GB treatment might reduce oxidative damage of membrane and ameliorate CI in papaya fruit. Similar result was also observed in GB‐treated sweet peppers and hawthorns (Razavi et al., 2018; Wang, Ding, Zuo, Gao, & Fan, 2016).

Previous studies suggest that loss of membrane integrity is closely related with energy depletion, in which low cellular energy status is responsible for limiting membrane lipid biosynthesis and accelerating lipid hydrolysis (Cao, Cai, Yang, Joyce, & Zheng, 2014). Liu et al. (2011) noted that pericarp browning in litchi fruit during room temperature storage after removal from refrigeration was a result of energy shortage and augmented activities of phospholipase D and lipoxygenase. In the present study, higher energy level as indicated by higher ATP content and energy charge was found in GB‐treated papaya fruit, which might account for maintenance of membrane integrity and reduced CI. Similar maintenance of energy status and membrane integrity in relation to CI alleviation was also reported in GB‐treated peaches and hawthorns (Razavi et al., 2018; Shan et al., 2016).

Regulation of energy status in plant tissues has involved energy metabolism‐related enzymes including ATPases, SDH, and CCO (Li et al., 2016). The mitochondrial membrane ATPases are a class of enzymes responsible for hydrolyzing ATP into ADP and phosphate, which contributes to production of more energy (Li et al., 2016). H^+^‐ATPase is an electrogenic enzyme that may couple ATP hydrolysis and pump‐out of H^+^ from the cell, and thus forming membrane electrochemical potential (Wang et al., 2015). Ca^2+^‐ATPase may regulate Ca^2+^ homeostasis by removing redundant Ca^2+^ from cytoplasm to extracellular matrix, which is essential for maintaining normal function of plant cells (Jin et al., 2013). Inhibition of the H^+^‐ATPase and Ca^2+^‐ATPase activities can result in damage of membrane integrity, suggesting that a sudden reduction in energization of plasma membrane might induce cell dysfunction (Jin et al., 2013). In the present study, higher activities of H^+^‐ATPase and Ca^2+^‐ATPase in parallel with alleviated CI were observed in GB‐treated papayas during low temperature storage, indicating that GB‐induced chilling tolerance might be related with enhancement of both ATPases activity. Similar results were also observed in methyl jasmonate (MeJA)‐treated peaches (Jin et al., 2013), NO‐ and H_2_S‐treated bananas (Li et al., 2016; Wang et al., 2015), 1‐methylcyclopropene‐treated pears (Cheng et al., 2015), and low temperature conditioned (LTC)‐loquats (Jin et al., 2015). These findings indicate that both H^+^‐ATPase and Ca^2+^‐ATPase in harvested crops could be favorable for affording tolerance to chilling stress by sustaining membrane energization (Aghdam et al., 2018).

Succinate dehydrogenase is the key enzyme of tricarboxylic acid cycle, which catalyzes the oxidation of succinate to fumarate and provides electron for respiratory chain, contributing to production of ATP in mitochondria (Liu et al., 2016). CCO is the last enzyme and the terminal electron acceptor in the mitochondrial respiratory electron‐transport chain, responsible for energy generation in mitochondria through oxidative phosphorylation (Jin et al., 2013). The activities of SDH and CCO are regarded as markers of energy metabolism and may reflect the functional status of mitochondria. The inactivation of SDH and CCO can lead to mitochondrial dysfunction as indicated by disturbed oxidative phosphorylation and reduced ATP generation, and consequently, resulting in CI occurrence (Jin et al., 2014). In the current study, higher SDH and CCO activities were observed in GB‐treated papaya fruit during cold storage when compared to untreated fruit, implying that GB could induce the increase in activity of key mitochondria enzymes involved in energy metabolism, thus contributing to elevated energy status and improved chilling tolerance. Similar results were also reported in MeJA‐treated peaches (Jin et al., 2013), H_2_S‐treated bananas (Li et al., 2016), and LTC‐treated loquats (Jin et al., 2015).

In summary, results in this study demonstrated that exogenous GB treatment at 15 mmol/L effectively ameliorated CI in papaya fruit during 40 days of refrigeration at 6°C. The improvement of chilling tolerance by GB might be ascribed to maintenance of membrane integrity under regulation of energy status as revealed by higher levels of ATP and energy charge. Furthermore, GB treatment resulted in increased activities of energy metabolism‐related enzymes including mitochondrial membrane H^+^‐ATPase and Ca^2+^‐ATPase, SDH, and CCO, which could benefit in elevating energy status and reducing CI in cold‐stored papaya fruit. However, underlying molecular mechanisms of GB‐mediated chilling tolerance in papaya fruit need further investigation. The present results also suggest that GB treatment could be an effective strategy to ameliorate CI in harvested papaya fruit during refrigeration.

## CONFLICT OF INTEREST

The authors declare that they do not have any conflict of interest.

## ETHICAL STATEMENT

This study does not involve any human testing.
